# Cancer Classification Based on Support Vector Machine Optimized by Particle Swarm Optimization and Artificial Bee Colony

**DOI:** 10.3390/molecules22122086

**Published:** 2017-11-29

**Authors:** Lingyun Gao, Mingquan Ye, Changrong Wu

**Affiliations:** 1 School of Medical Information, Wannan Medical College, Wuhu 241002, China; anningxia55@163.com; 2 School of Mathematics and Computer Science, Anhui Normal University, Wuhu 241002, China; wcr193@126.com

**Keywords:** intelligent optimization, cancer classification, PSO, ABC, SVM

## Abstract

Intelligent optimization algorithms have advantages in dealing with complex nonlinear problems accompanied by good flexibility and adaptability. In this paper, the FCBF (Fast Correlation-Based Feature selection) method is used to filter irrelevant and redundant features in order to improve the quality of cancer classification. Then, we perform classification based on SVM (Support Vector Machine) optimized by PSO (Particle Swarm Optimization) combined with ABC (Artificial Bee Colony) approaches, which is represented as PA-SVM. The proposed PA-SVM method is applied to nine cancer datasets, including five datasets of outcome prediction and a protein dataset of ovarian cancer. By comparison with other classification methods, the results demonstrate the effectiveness and the robustness of the proposed PA-SVM method in handling various types of data for cancer classification.

## 1. Introduction

Owing to no obvious early symptoms of cancer, most of patients are diagnosed at an advanced stage [1], which usually results in high costs with a poorer prognosis. In addition to uncontrolled growth, cancer cells invade the surrounding normal tissue and even move through the body’s circulatory system or lymphatic system to other parts of the body [2]. The probability of recurrence or metastasis after surgery is higher than 90% after five years, as cancer treatment is not thorough. To date, the problem of completely clearing the remaining cancer cells is not solved, and the recurrence rate and mortality rate of cancer are still quite high. If we can make full use of available human expression profiles and realize repeatable diagnoses, there is no doubt that it will bring great convenience to cancer patients. The analysis of microarray gene expression data [3] and protein expression data can be used to grasp the information of physiological activities at the molecular level, which is widely used in the field of biomedicine. However, a large number of irrelevant and redundant values exist in expression profiles. Moreover, the high dimensionality and small sample bring great difficulties to the data processing. Thus, researchers have proposed various methods [4,5,6,7,8] to deal with these problems.

Up to now, many machine learning algorithms have been applied to the classification study of bioinformatics, such as random forest [9], k-nearest neighbor, neural network [10], and SVM (Support Vector Machine). Besides these, there are also some ensemble classifiers [11,12,13,14]. Among them, DNA recombination spots were identified by a web server iRSpot-EL based on ensemble learning [13]. A two-layer ensemble classifier named 2L-piRNA makes it possible to efficiently identify piwi-interacting RNAs and their function [14]. In addition, the classifier was operated with the SVM algorithm. Another method based on the SVM model is iEnhancer-2L, which is a two-layer predictor for identifying enhancers and their strength [15]. SVM has distinctive advantages in handling data with high dimensionality and a small sample size. In addition, nonlinear data can be mapped into high dimensional space rely on kernel function, and turned into linear separable problem [16]. The kernel function of RBF (Radial Basis Function) [17] is widely used due to its superiority in parameter and classification performance. Notably, the choice of penalty factor C and kernel parameter σ of SVM affects classification results. Many researchers have used a variety of search methods to find the optimal parameters [18,19,20,21]. At the same time, the parameters of SVM have been optimized by many intelligent algorithms in Reference [21].

Intelligent optimization algorithms are developed by simulating or revealing some natural phenomena, and are widely used in many research fields because of their versatility [22,23,24,25]. The PSO (Particle Swarm Optimization) algorithm has been successfully applied to cancer classification because of its simplicity and generality [26]. However, PSO is easily falls into the local optimal solution. In addition, the ABC (Artificial Bee Colony) algorithm possesses good global convergence and extensive applicability [27]. However, ABC is unsatisfactory at exploitation [28]. The use of a single optimization algorithm presents the shortcomings of low precision and weak generalization ability in solving complex problems. To further explore the application of intelligent optimization in bioinformatics, PSO and ABC are combined in this paper, which means the ability of exploitation and exploration are combined for binary and multiclass cancer classification. In this paper, the FCBF (Fast Correlation-Based Feature selection) method [29] is employed to remove the redundant and irrelevant features, the PSO optimization results are taken as the initial values of ABC, and then the cancer classification model is constructed after the parameters are tuned; this hybrid method is represented as PA-SVM. Nine cancer datasets are utilized for experiments, and then compared with other classification methods: LIBSVM (A Library for Support Vector Machine), GA-SVM (Genetic Algorithm combined with SVM), PSO-SVM (Particle Swarm Optimization combined with SVM), and ABC-SVM (Artificial Bee Colony combined with SVM). The results demonstrate that the proposed PA-SVM method presented in this paper has good robustness and achieves more accurate classification results. This work aims to provide cancer classification results for reference and to make a contribution to the clinical diagnosis and treatment of different types of cancer.

## 2. Results

### 2.1. Feature Selection

Within nine cancer datasets, the number of features ranges from 2000 to tens of thousands; the breast cancer dataset being the largest with 24,481 attributes. Since a large number of irrelevant and redundant attributes are involved in these expression data, the cancer classification task is made more complicated. If the complete data is used to perform cancer classification, the accuracy will be not so accurate, and high computational time and cost will be incurred. Therefore, the reliable FCBF method [29] is adopted to select a subset of discriminatory features before classification. By discarding the attributes with little or no effect, FCBF provides good performance with full consideration of feature correlation and redundancy. In this paper, we standardized the data first, and then performed the feature selection by FCBF in Weka. The number of reduced attributes is shown in Table 1.

As shown from this table, the number of attributes subtracted is far less than the original number. Among them, the number of attributes of breast cancer decreased from 24,481 to 92, while the number of attributes of lung cancer decreased from 2880 to 6. Furthermore, the proteomic spectra of ovarian cancer include 30 reduced attributes, while the original number was 15,154. Consequently, the number of original features is reduced by a large amount, and the computational cost is reduced as well.

### 2.2. Cancer Classification

The classification experiment in this paper was carried out under a Matlab environment. Nine subsets after feature reduction were used as input data of the proposed PA-SVM classifier for classification. In order to verify the effectiveness of the proposed method, we compared it with the LIBSVM method of default parameters in Weka, as well as with GA-SVM, PSO-SVM, and ABC-SVM in Matlab. Among these techniques, SVM used in this experiment is derived from the LIBSVM package developed by Prof. Chih-Jen Lin. GA-SVM used genetic algorithm (GA) to optimize SVM parameters and applied the optimized SVM to classify cancer datasets. GA has the characteristics of parallel computing, which is suitable for large-scale complex problem optimization. PSO-SVM utilized PSO to optimize the parameters of SVM. PSO is easy to implement with few parameters to adjust, while it also has advantages of fast convergence speed and strong versatility. ABC-SVM employed ABC to find the optimal parameters of SVM, which takes full advantage of the good global convergence and flexibility of ABC. Therefore, the PA-SVM algorithm proposed in this paper can combine the exploitation of PSO and the exploration of ABC, and overcome the disadvantages of PSO easily falling into local optimum as well as the weak exploitation of ABC. In addition, due to the small number of selected features, 10-fold cross validation was used. For the purpose of avoiding instable operation results, each experiment was run 10 times, and the optimal classification accuracy was selected for comparison. The final classification results are shown in Table 2.

As seen from Table 2, the accuracy of the last column, which represents the classification result of the proposed PA-SVM method, always obtains the optimal values. Among these cancer datasets, for the five groups of patients with outcome prediction, such as breast cancer and nervSys, the proposed PA-SVM method achieved the same accuracy as ABC-SVM, and both were higher than other methods. As for lung cancer, the best result was obtained by PA-SVM and PSO-SVM. This showed that the PA-SVM approach described in this paper has superior outcome prediction. To elaborate, the leukemia dataset contains three disease types, and all of these methods can achieve 100% classification accuracy. Concerning the protein data of ovarian cancer, the constructed classification model obtained 100% accuracy. This study is significant for women who have a high risk of ovarian cancer due to a family or personal history of cancer. With regard to DLBCL1 and DLBCL2, the proposed PA-SVM achieved the highest performance compared to other classification approaches.

**Figure 1:**
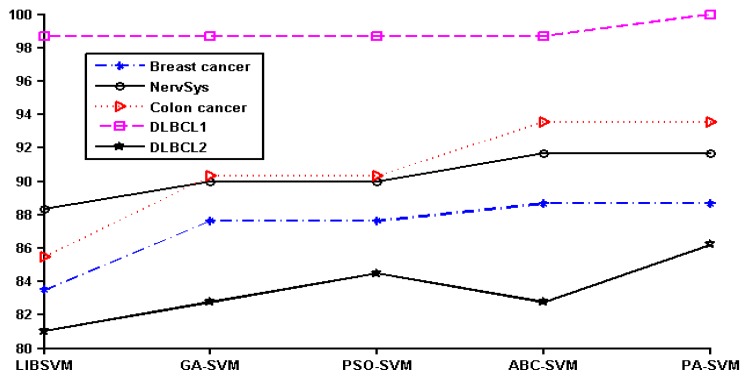
Cancer classification accuracy (%) of different methods.

In order to show the effect of various methods intuitively, we converted the data in Table 2 to a line chart. Since the classification accuracy for the prostate, leukemia, and ovarian datasets was 100%, they were not shown in the figure. The details of the remaining six datasets can be seen in Figure 1.

It can be seen that PA-SVM has the optimal accuracy for all cancer datasets studied. Also, in the experiments it can be observed that ABC-SVM and PA-SVM maintain better robustness, while GA-SVM and PSO-SVM easily fall into the local optimal solution. Accordingly, the proposed PA-SVM approach always obtains the best classification performance and has good robustness. This indicates that PA-SVM is able to handle multiple types of data with better performance in cancer prognosis, identification, and classification compared to other methods employed.

## 3. Discussion

The complexity and variability of cancer greatly increases the difficulty of clinical treatment. In addition, patients still have a large probability of recurrence after treatment, which is still a serious threat to people’s health. Thus, the early diagnosis and prognosis of cancer is of great significance. However, there are many problems during the handling of gene expression data, such as high dimension, small sample size, and large noise. In order to obtain truly valuable genes, a variety of methods are proposed for feature selection and classification prediction.

Intelligent optimization algorithms have advantages of fast convergence, simple operation, adaptability, and robustness, and they are applied in many fields. In this paper, the FCBF method was used to filter the irrelevant and redundant attributes, and the obtained subsets were input into the constructed classification model based on PSO and ABC. In this paper, the optimal solution obtained by PSO is taken as the initialization of the ABC algorithm, and thus the advantages of PSO and ABC were combined to constitute the PA-SVM classifier. It has been observed that the proposed method can always get the best results through the classification of different cancer datasets with a low number of selected genes. In addition, this paper also used ovarian cancer protein expression data for cancer classification because of the importance of protein molecules. The results demonstrated that the proposed PA-SVM method has greater advantages for cancer prognosis, identification, and classification, as well as the handling of multiple types of datasets.

The causes of cancer are full of complexity and variety; therefore, single type of data probably not provides an adequate explanation or makes a perfect prediction. We will thus combine multiple types of data in the future in order to achieve a comprehensive analysis. In addition, the theoretical mechanism of intelligent optimization still lacks a complete mathematical foundation. Further study and in-depth research are needed. At the same time, clinical validation is also necessary in order to make the research more conducive to human life.

## 4. Materials and Methods

### 4.1. Cancer Datasets

The datasets used in the experiment were all derived from the Kent Ridge Bio-medical Dataset Repository, mentioned in Reference [30]. These datasets include breast cancer, colon cancer, DLBCL, lung cancer, leukemia, nervSys (central nervous system embryonal tumor), ovarian cancer, and prostate cancer. A detailed description of these datasets is shown in Table 3.

In addition to colon, DLBCL1, leukemia, and ovarian datasets, the remaining datasets concerned the outcome prediction of cancer patients, indicating a predicted relapse or non-relapse. It is worth noting that the ovarian cancer measures protein expression data. The goal of this experiment was to identify proteomic patterns in serum that distinguish ovarian cancer from non-cancer. Among them, the DLBCL dataset included two categories: DLBCL1 and DLBCL2. The first is DLBCL versus Follicular Lymphoma (FL) morphology. The second is the outcome prediction data of 58 cases of DLBCL samples, some of them from cured patients and others from patients with fatal or refractory disease. Besides DLBCL, the colon cancer dataset contained 62 samples collected from colon cancer patients, characterized by a common malignant tumor in the gastrointestinal tract. Also, the leukemia dataset of 72 samples included three categories: ALL, MLL, and AML. They stand for acute lymphoblastic leukemia, mixed lineage leukemia, and acute myeloid leukemia, respectively.

### 4.2. Particle Swarm Optimization (PSO) Algorithm

**Figure 2:**
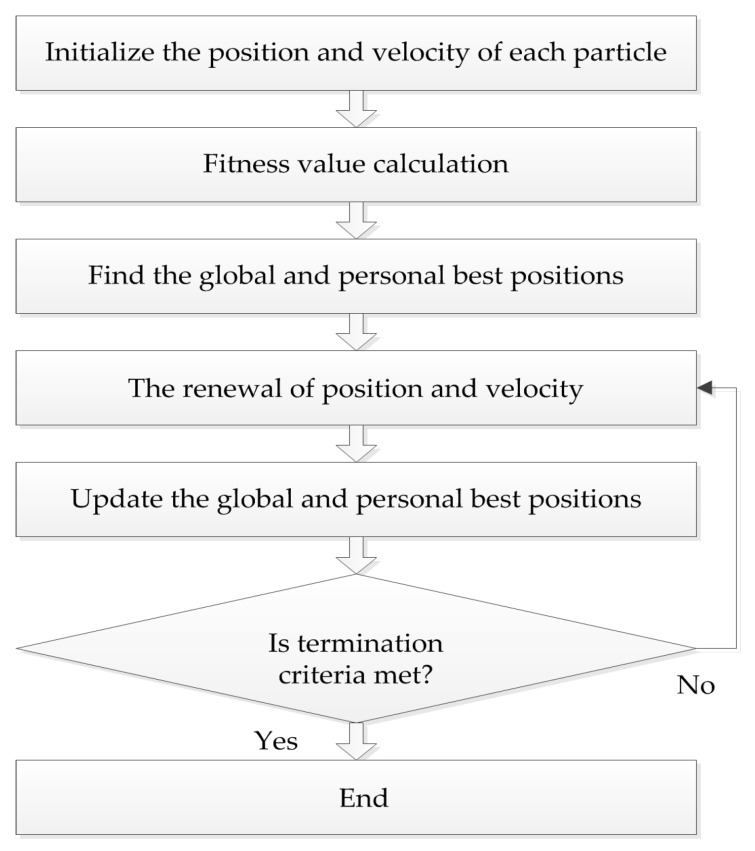
The flowchart of PSO algorithm.

The PSO algorithm originated from the study of bird predation behavior, first proposed by Kennedy and Eberhart in 1995. The algorithm is easy to implement and the rules are simple. The position of each particle represents a potential solution, each particle has a fitness value determined by fitness function, and each particle has three characteristics: position, velocity, and fitness value. The flowchart of the PSO algorithm is shown in Figure 2.

Suppose that in the feasible solution *D*-dimensional space, the population of *n* particles is *X* = [*X*_1_, *X*_2_, …, *X_n_*]. The position and velocity of the *i*-th particle are *X_i_* = [*x_i_*_1_, *x_i_*_2_, …, *x_iD_*] and *V_i_* = [*v_i_*_1_, *v_i_*_2_, …, *v_iD_*], respectively, where the velocity determines the direction and distance of the particle movement. The particles move in the feasible solution space, and the individual position is updated by tracking the personal best position *P_i_* and the global best position *P_g_.* The personal best position is the best location with best fitness value experienced by the individual: *P_i_* = [*p_i_*_1_, *p_i_*_2_, …, *p_iD_*]. The global best position is the optimal position with best fitness for all particles in the population: *P_g_* = [*p_g_*_1_, *p_g_*_2_, …, *p_gD_*]. The particle updates velocity and position through the global and personal best positions. The formula is represented as:

(1) $$\begin{array}{l} v_{id}^{k+1}=\omega v_{id}^{k}\;+\;c_{1}r_{1}(p_{id}^{k}\;-\;x_{id}^{k})\;+\;c_{2}r_{2}(p_{gd}^{k}\;-\;x_{id}^{k})\\ \;d=1,\;2,\;\ldots ,\;D \end{array}$$

(2) $$\begin{array}{l} x_{id}^{k+1}=x_{id}^{k}+v_{id}^{k+1}\\ \;d=1,2,\ldots ,D;\;i=1,\;2,\;\ldots ,\;n \end{array}$$

where *k* is the current iteration number, *ω* is the inertia weight, *c*_1_ and *c*_2_ denote the cognitive and the social learning factors, and *r*_1_ and *r*_2_ are uniformly distributed numbers in the interval (0, 1). In order to avoid a blind search, the position and velocity of each particle has a certain limit interval [*X*_min_, *X*_max_] and [*V*_min_, *V*_max_].

### 4.3. Artificial Bee Colony (ABC) Algorithm

**Figure 3:**
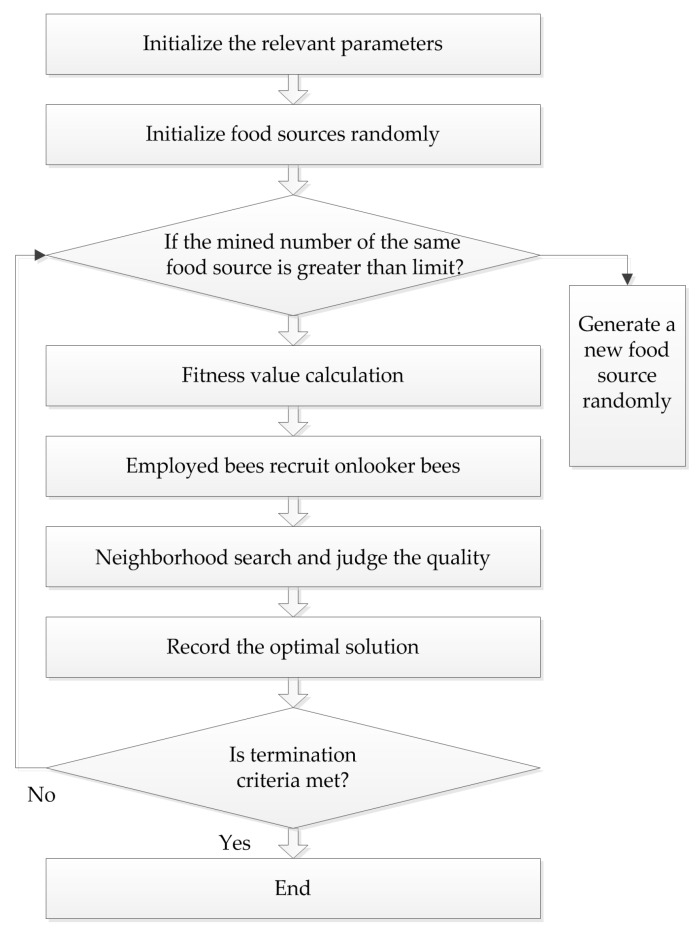
The flowchart of the ABC algorithm.

Karaboga successfully applied the bee colony algorithm to the extreme value optimization problem in 2005. At the same time, he systematically put forth the ABC algorithm, which simulates bees gathering nectar in nature. In this algorithm, bees are divided into three types: employed bees, onlooker bees, and scout bees, and they find the optimal solution through the collection and sharing of nectar. The procedure of the ABC algorithm is presented in Figure 3.

In the ABC algorithm, the position of each food source represents a feasible solution of the problem to be optimized. The enrichment of each source corresponds to the fitness of each solution. First, feasible solution *x_i_* is initialized in the *D*-dimensional space, and *i* = 1, 2, …, *N*, each food source attracts one employed bee, so the position of the source is the position of the employed bee. Onlooker bees determine the location according to the selection probability of the fitness value that employed bees remembered:

(3) $$P_{i}=\frac{fit_{i}}{\sum_{n=1}^{N}fit_{n}}$$

where *fit_i_* is the fitness of the *i*-th food source, and *P_i_* is the selection probability. Then, onlooker bees do the neighborhood search according to the formula:

(4) $$x_{i}^{k+1}=x_{i}^{k}+r\times (x_{i}^{k}-x_{j}^{k})$$

where *i* ≠ *j*, *j* = 1, 2,…, *N*, and $x_{i}^{k}$ is the current food source position, and *r* is a random number in the range of (–1, 1). Subsequently, the fitness values before and after the search are compared, and the better one is chosen. If the source has not been changed by multiple searches, and the number of times exceeds the limit, the employed bees’ identity will become scout bees, and another solution is generated randomly. The formula for producing a new food source is as follows:

(5) $$x_{i}=x_{\min}+rand(0,\;1)(x_{\max}-x_{\min})$$

This method not only improves the quality of the solution, but also increases the diversity, which is beneficial to finding the global optimal solution.

### 4.4. The Proposed PA-SVM Algorithm

**Figure 4:**
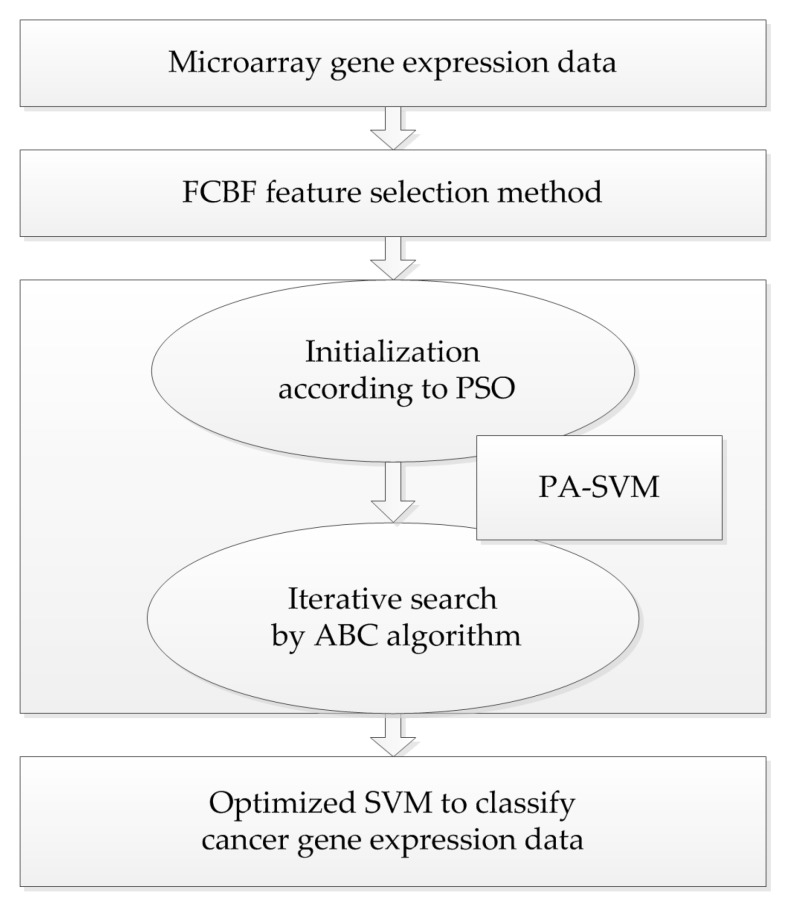
The whole frame of the proposed PA-SVM algorithm.

Taking into account the large number of irrelevant and redundant values in gene expression data, we utilized the FCBF feature selection method [26] combined with SymmetricalUncertAttributeSetEval attribute evaluator in Weka to select gene subsets, and these subsets were input into the constructed cancer classification model for classification evaluation. The whole process of the experiment is shown in Figure 4.

SVM finds the classification interval of two kinds of samples as large as possible by looking for an optimal classification hyperplane that satisfies the requirements. When dealing with non-linear samples, kernel function is used to map the data into a high-dimensional space, so that the data can be linearly separable. The search for the optimal hyperplane can be converted to the minimization of the following equation by introducing the penalty factor *C* and slack variable *ξ*.

(6) $$\begin{array}{l} f=\frac{1}{2}{\left\|\omega \right\|}^{2}+C\sum_{i=1}^{N}\xi_{i}\\ s.t.\left\{ \begin{array}{l} \;y_{i}(\omega^{T}\phi (x_{i})+b)\geq 1-\xi_{i}\\ \;\xi_{i}\geq 0 \end{array}\right. \end{array}$$

where *ω* is the hyperplane normal vector, and *b* is the threshold. The feature map *ψ*(*x*) is a kernel function that satisfies the Mercer condition. Since this paper adopts the widely used RBF kernel function, its value is mainly affected by the kernel function parameter *σ.* It can be seen that the penalty factor *C* and the kernel function parameter *σ* have a great influence on the classification performance of SVM.

This article uses the easy-to-use LIBSVM package developed by professor Chih-Jin Lin of Taiwan University, which can solve the complex problem of multi-class pattern recognition. The proposed method PA-SVM employs PSO and ABC to optimize the penalty factor C and parameter σ of RBF kernel function in SVM.

The personal and global best values are determined by making full use of the optimal value in the optimization process and the introduction of mutation, which gives PSO good exploitation abilities. The solutions of parameters bestc and bestg found by PSO are used as the initial values of food sources in the ABC algorithm, thus the uncertainty of random values can be avoided to some extent. Bestc means the best value of parameter C found by PSO, while bestg is the best value of the RBF kernel function parameter. Then, as PA-SVM takes full use of the exploration of scout bees in ABC, it is more conducive to discover the global optimum by overcoming the shortcoming of PSO, which is easily trapped in the local optimal. This method combines the advantages of the PSO and ABC algorithms by striking a balance between exploration and exploitation.

The higher classification accuracy is the main purpose of optimizing SVM, and the fitness function in this paper is directly represented as the value of classification accuracy, which is expressed as follows:

(7) $$F=V_{accuracy}$$

In this study, the dimension parameter *D* is 2, the population size is set to 30, the number of iterations is 100, the limit is 50, and a 10-fold cross validation method is used to assess the classification accuracy.

## Figures and Tables

**Table 1:** Reduced attributes by FCBF.

Datasets	Original Attributes	Reduced Attributes
Breast cancer	24,481	92
Lung cancer	2880	6
NervSys	7129	28
Prostate cancer	12,600	27
Colon caner	2000	14
Leukemia	12,582	97
Ovarian cancer	15,154	30
DLBCL1 ^1^	7129	73
DLBCL2	7129	27

^1^ DLBCL represents diffuse large B-cell lymphoma.

**Table 2:** Classification accuracy of different methods.

Datasets	No. of Attributes	LIBSVM (%)	GA-SVM ^1^ (%)	PSO-SVM ^2^ (%)	ABC-SVM ^3^ (%)	PA-SVM ^4^ (%)
Breastcancer	92	83.51	87.63	87.63	**88.66**	**88.66**
Lung cancer	6	64.10	66.67	**79.49**	74.36	**79.49**
NervSys	28	88.33	90	90	**91.67**	**91.67**
Prostate cancer	27	90.48	**100**	**100**	**100**	**100**
Colon caner	14	85.48	90.32	90.32	**93.55**	**93.55**
Leukemia	97	**100**	**100**	**100**	**100**	**100**
Ovarian cancer	30	**100**	**100**	**100**	**100**	**100**
DLBCL1	73	98.70	98.70	98.70	98.70	**100**
DLBCL2	27	81.03	82.76	84.48	82.76	**86.21**

^1^ GA-SVM method is genetic algorithm combined with SVM; ^2^ PSO-SVM denotes particle swarm optimization combined with SVM; ^3^ ABC-SVM means artificial bee colony method is used to optimize SVM; ^4^ PA-SVM combines particle swarm optimization with artificial bee colony to optimize SVM. The bold in the table represents the optimal value.

**Table 3:** Details of cancer datasets.

Datasets	Samples	No. of Attributes	Classes	Labels
Breast cancer	97	24,481	2	outcome prediction
Lung cancer	39	2880	2	outcome prediction
NervSys	60	7129	2	outcome prediction
Prostate cancer	21	12,600	2	outcome prediction
Colon caner	62	2000	2	cancer or not
Leukemia	72	12,582	3	multi-category
Ovarian cancer	253	15,154	2	protein data
DLBCL1	77	7129	2	two category
DLBCL2	58	7129	2	outcome prediction
